# A Lightweight Semantic Segmentation Model for Underwater Images Based on DeepLabv3+

**DOI:** 10.3390/jimaging11050162

**Published:** 2025-05-19

**Authors:** Chongjing Xiao, Zhiyu Zhou, Yanjun Hu

**Affiliations:** School of Computer Science and Technology, Zhejiang Sci-Tech University, Hangzhou 310018, China

**Keywords:** semantic segmentation, lightweight network, attention mechanism, strip pooling, feature fusion

## Abstract

Underwater object image processing is a crucial technology for marine environmental exploration. The complexity of marine environments typically results in underwater object images exhibiting color deviation, imbalanced contrast, and blurring. Existing semantic segmentation methods for underwater objects either suffer from low segmentation accuracy or fail to meet the lightweight requirements of underwater hardware. To address these challenges, this study proposes a lightweight semantic segmentation model based on DeepLabv3+. The framework employs MobileOne-S0 as the lightweight backbone for feature extraction, integrates Simple, Parameter-Free Attention Module (SimAM) into deep feature layers, replaces global average pooling in the Atrous Spatial Pyramid Pooling (ASPP) module with strip pooling, and adopts a content-guided attention (CGA)-based mixup fusion scheme to effectively combine high-level and low-level features while minimizing parameter redundancy. Experimental results demonstrate that the proposed model achieves a mean Intersection over Union (mIoU) of 71.18% on the DUT-USEG dataset, with parameters and computational complexity reduced to 6.628 M and 39.612 G FLOPs, respectively. These advancements significantly enhance segmentation accuracy while maintaining model efficiency, making the model highly suitable for resource-constrained underwater applications.

## 1. Introduction

Underwater image semantic segmentation is critical for advancing marine exploration, habitat monitoring, and autonomous underwater operations. However, the aquatic environment introduces unique challenges: light attenuation and scattering in water cause severe color distortion, while suspended particles and turbidity degrade image contrast, resulting in blurred textures and ambiguous object boundaries. These degradation effects not only obscure critical visual cues but also amplify the complexity of feature extraction, rendering conventional segmentation models—often optimized for terrestrial imagery—ineffective in underwater scenarios.

Recent advances in the field of image segmentation leverage deep learning to deal with complex scenes. The atrous convolution in DeepLab [1] has shown its effectiveness in extracting multi-scale contextual features, and Fully Convolutional Networks [2] provide a unified framework for pixel-level prediction. The integration of dynamic kernel mechanisms in K-Net [3] enables adaptive feature refinement for different segmentation tasks, and Transformer-based DETR [4] provides an end-to-end paradigm by detecting and segmenting objects in a unified way. Asymmetric Non-local Networks [5] effectively model long-range spatial dependencies and reduce the computational overhead.

With the growing demand for underwater machine vision, semantic segmentation of underwater images has emerged as a critical research field. Liu et al. [6] developed an underwater semantic segmentation network with an unsupervised color correction module to improve input image quality. Zhou et al. [7,8,9] proposed a suite of underwater image enhancement techniques to address diverse degradation issues. Islam et al. [10] introduced SUIM, the first large-scale dataset for underwater semantic segmentation. WaterBiSeg-Net [11] performs real-time segmentation of marine garbage by enhancing and suppressing background information with multi-scale information. UIE-Convformer [12] is a fusion CNN and feature fusion Transformer network that uses a multi-scale U-Net, local and global feature extraction, multi-scale fusion, and refinement modules to obtain better segmentation effects. CEWformer [13], a transformer-based network that enables image enhancement and watermarking at the same time, provides a new way to think about underwater segmentation. However, most existing models overlook lightweight design, prompting increasing attention to efficient architectures. ENet [14] reduced model complexity but suffered from limited receptive fields. OCNet [15] integrated cross-sparse self-attention with dilated pyramid pooling to boost accuracy. Separable convolution-based networks minimized computational load, while CGNet [16] simultaneously learned local and global features with under 0.5 M parameters, albeit with compromised accuracy. DFANet [17] proposed deep feature aggregation to combine network-level and stage-level representations, while LEDNet [18] accelerated processing via a lightweight upsampling module. BiseNetv2 [19] adopted a two-branch architecture, where the semantic branch outputs guided spatial branch features to improve performance. Despite these advancements in terrestrial domains, underwater segmentation remains challenging due to the low contrast, high noise, and inherent blurriness in underwater imagery, which often result in rough edges and ambiguous semantic boundaries in segmentation outputs. Furthermore, real-time processing demands for resource-constrained platforms, such as autonomous underwater vehicles (AUVs) or low-power embedded systems, necessitate models that balance accuracy with computational efficiency.

To solve the above problems, this paper proposes a lightweight semantic segmentation network for underwater scenarios and conducts experiments on the underwater semantic segmentation dataset DUT-USEG [20]. This study selects the MobileOne-S0 [21] network as the main feature extractor based on the lightweight architecture design criteria. Through structural reparameterization technology, this network constructs a multi-branch topology during the training phase to enhance feature representation capabilities and merges into a single branch during inference, thus achieving efficient feature encoding and optimization of computational costs. To further enhance the multi-scale feature expression ability, SimAM [22] is innovatively introduced. This module constructs three-dimensional attention weights based on the energy function theory, adaptively enhances the saliency feature responses in the target area by implicitly modeling the feature correlations in the channel and spatial dimensions, and does not introduce additional learnable parameters. Aiming at the limitations of the global information capture of the ASPP module, the strip pooling [23] strategy is adopted to replace the traditional global average pooling. By constructing long and narrow pooling kernels in the horizontal and vertical directions, the long-range context dependencies of the image are extracted simultaneously, effectively broadening the global receptive field of the model. In the high-level and low-level features fusion stage, a mixup fusion scheme based on CGA [24] is introduced. By dynamically calibrating the receptive fields and semantic consistency of features at different levels, it suppresses information redundancy in cross-scale feature interactions and achieves efficient aggregation of feature maps.

Experimental data show that the improved model reaches an mIoU index of 71.18% on the DUT-USEG dataset, while maintaining a computational complexity of 6.628 M parameters and 39.612 GFLOPs. Under the premise of ensuring the lightweight nature of the model, this scheme provides an effective technical implementation path for real-time semantic segmentation tasks of underwater biological images.

The rest of the paper is organized as follows: Section 2 describes the materials and methods in detail; Section 3 analyzes the experimental results and is followed by the conclusion in Section 4.

## 2. Materials and Methods

### 2.1. DeepLabv3+ Model

DeepLabv3+ stands out as an advanced semantic segmentation model, serving as an enhanced version of DeepLabv3 with a residual network typically employed as the underlying architecture. It incorporates the ASPP module and an encoder–decoder framework. The encoding module comprises a backbone feature extraction network—often mainstream architectures such as Xception, ResNet, MobileNetV2, and ShuffleNet—and the ASPP module. The DeepLabv3+ model first performs feature learning, optimization, and hierarchical representation through the backbone network to generate coarse feature maps. These coarse maps are then processed by ASPP to extract and aggregate multi-perspective contextual information. The decoding module fuses low-level features with high-level features, integrating pixel-level location details with contextual information, and employs bilinear interpolation for upsampling to achieve pixel-wise segmentation. By leveraging depthwise separable convolution (DSC) and dilated/atrous convolution (DC), DeepLabv3+ effectively increases network depth while keeping model parameters in check.

### 2.2. Methodology

#### 2.2.1. MobileOne-S0

Many current improvements to the DeepLabv3+ model involve replacing the relatively “heavyweight” backbone network with the classic lightweight backbone network MobileNetV2. The primary reason for this is MobileNetV2’s own 3.4 M parameters and 0.98 ms inference latency, which bring significant performance advantages. After replacement, the DeepLabv3+ model also demonstrates excellent performance. While MobileNetV2 brings competitiveness, it also has several shortcomings, mainly in the following three aspects: first, the alternating stacking of depthwise convolutions and linear bottleneck structures limits the effective receptive field; second, the channel splitting mechanism of depthwise separable convolutions increases computational redundancy for cross-channel information interaction; more critically, although the adopted H-swish activation function can enhance representation capabilities, it incurs significant memory access overhead in mobile hardware or embedded device acceleration. These design flaws make it difficult for MobileNetV2 to meet higher efficiency requirements in latency-sensitive scenarios.

**Figure 1:**
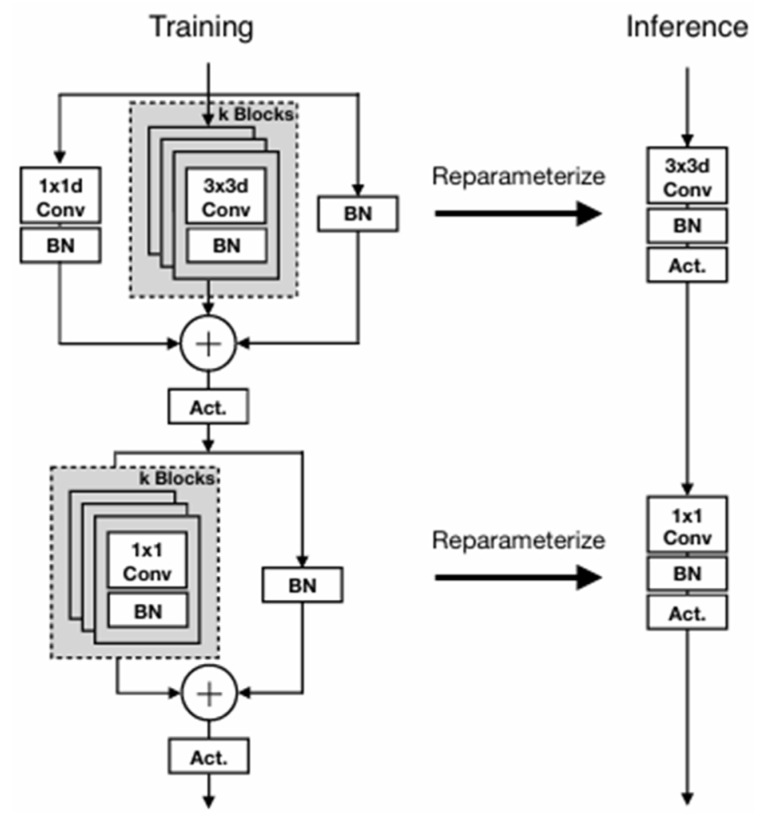
MobileOne Block.

As a representative of newer lightweight network architectures, MobileOne-S0 demonstrates significant advantages in efficient computing scenarios for mobile and embedded devices. Figure 1 illustrates the structural differences in the core modules of MobileOne during the training and inference phases. During training, the module employs a multi-branch design: the main branch consists of 3 × 3 depthwise separable convolutions, while introducing reparameterizable skip connections (including 1 × 1 convolutions and batch normalization layers) and multiple trivial over-parameterized branches (controlled by the hyperparameter k). These branches enhance the model’s feature expression capabilities through parameter sharing. During inference, all branches are merged into a single linear structure via structural reparameterization techniques, eliminating multi-branch operations and forming a directly connected network without skip connections (as shown in the right half of Figure 1). This design significantly reduces computational latency on mobile devices while preserving the multi-scale feature learning capabilities of the training phase.

Table 1 specifically defines the network hierarchy configurations of different MobileOne variants (S0–S4). As shown in the table, the network is divided into 8 stages. Early stages (e.g., Stages 1–2) process high-resolution inputs (224 × 224 to 112 × 112) and use fewer blocks (1–2 blocks) to reduce computational load. Deep stages (e.g., Stages 3–5) densely stack blocks on low-resolution feature maps (e.g., Stage 3 contains 8 blocks), with channel numbers dynamically adjusted by the width scaling coefficient α (e.g., α = 0.75 for S0, α = 3.0 for S4). This progressive complexity strategy introduces SE-ReLU activation functions in Stages 5–6 (only used in the largest variant S4) and replaces convolutional operations with global average pooling in Stage 7 to further optimize computational efficiency. By comparing the parameter settings of different variants, MobileOne achieves model scaling by adjusting channel numbers rather than input resolution, avoiding the sharp increase in FLOPs and memory consumption caused by resolution improvements. To achieve the lightest weight, this study selects MobileOne-S0 as the backbone network from various MobileOne variants.

#### 2.2.2. SimAM

**Figure 2:**
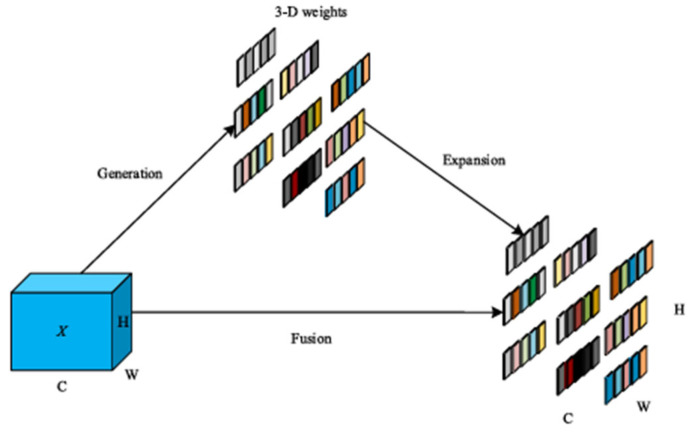
The structure of SimAM.

The ASPP in DeepLabv3+ extracts multi-scale context information from high-level features. Applying SimAM after ASPP further refines these features. It strengthens the correlations among different multi-scale features obtained by ASPP in both spatial and channel dimensions. Without adding extra parameters, SimAM generates more discriminative three-dimensional attention weights. This enables the model to better distinguish and utilize the multi-scale context information provided by ASPP, thus improving the accuracy of object segmentation, especially for objects of diverse scales. The structure of the SimAM attention mechanism is shown in Figure 2.

Using relevant theories of neural networks, relatively important features are selected to achieve semantic segmentation of images. The energy function is defined as

(1) $$e_{t}\left(w_{t},b_{t},y,x_{i}\right)=(y_{t}-\hat{t})+\frac{1}{M-1}\sum_{i=1}^{M-1}{\left(y_{o}-\hat{x_{i}}\right)}^{2}$$

In Equation (1), $\hat{t}=w_{t}t+b_{t}$, $\hat{x_{i}}=w_{t}x_{i}+b_{t}$, and binary labels are introduced. t and $x_{i}$ represent the input features of the target neuron and other neurons, respectively, while $w_{t}$ and $b_{t}$ denote the weights and biases of the linear transformation. M is the number of neurons in a channel. $w_{t}$ and $b_{t}$ can also be expressed by the following equations:

(2) $$w_{t}=-\frac{2(t-u_{t})}{{\left(t-u_{t}\right)}^{2}+2{\sigma_{t}}^{2}+2\lambda }$$

(3) $$b_{t}=-0.5(t+u_{t})w_{t}$$

where t denotes the regularization coefficient, and $u_{t}$ and ${\sigma_{t}}^{2}$ represent the mean and variance of neurons, respectively. After Equation (1) is minimized, a regularization term is introduced to obtain the final energy function:

(4) $$e_{t}^{*}=\frac{4\left({\hat{\sigma }}^{2}+\lambda \right)}{{\left(t-u_{t}\right)}^{2}+2{\sigma_{t}}^{2}+2\lambda }$$

Equation (4) indicates that the smaller the value of the energy function, the more important it is. Therefore, the saliency can be evaluated by the reciprocal of the neuron, and the features can be enhanced through normalization by the Sigmoid function. SimAM is a lightweight, parameter-free attention module designed to enhance feature discriminability in deep networks. Rooted in energy function theory, it dynamically generates 3D attention weights (channel and spatial) by analyzing feature statistics to suppress background noise and highlight salient target regions. Integrated after the ASPP module in DeepLabv3+, SimAM refines multi-scale contextual features without introducing additional learnable parameters. By adaptively emphasizing relevant features, it improves the model’s ability to distinguish objects of varying scales in complex underwater scenes.

#### 2.2.3. Strip Pooling

**Figure 3:**
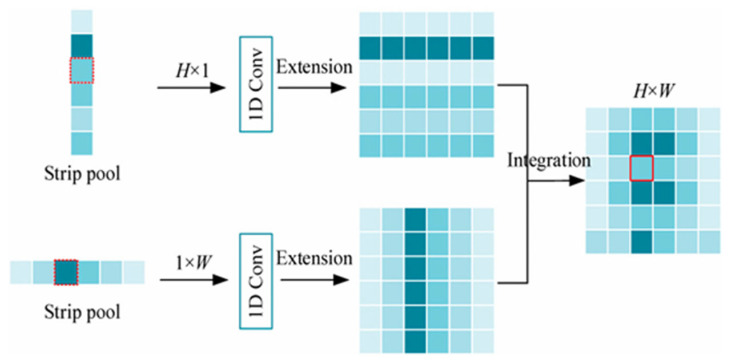
The structure of strip pooling.

Replacing global average pooling with strip pooling in DeepLabv3+’s ASPP module addresses two limitations of global average pooling while preserving multi-scale context aggregation. Unlike global average pooling, which compresses global features into a single vector and risks oversmoothing spatially anisotropic structures, strip pooling employs horizontal and vertical long-kernel pooling to explicitly model long-range dependencies along orthogonal axes. Moreover, when combining the two spatial dimensions, the feature values of rows or columns are weighted averages. This complements ASPP’s multi-scale atrous convolutions by adding direction-aware contextual priors, particularly beneficial for segmenting irregularly shaped targets. Strip pooling retains global context via its hybrid local–global receptive fields, enhancing feature discriminability without sacrificing computational efficiency. This adaptation synergizes with ASPP’s core design philosophy of multi-scale fusion while addressing the spatial bias inherent to isotropic pooling operations. The model structure is shown in Figure 3 below. For the input image, the calculation equation for the output of the row vector is as follows:

(5) $$y_{i}^{h}=\frac{1}{W}\sum_{0\leq j<w}X_{i,j}$$

And the calculation equation for the column vector output is as follows:

(6) $$y_{i}^{v}=\frac{1}{H}\sum_{0\leq i<H}X_{i,j}$$

For the input feature map $X\in R^{H\times W\times C}$, *H* and *W* denote the height and width, and *C* denotes the number of channels. Pooling is performed on *X* in the horizontal and vertical directions, and the resulting outputs are $y^{h}\in R^{H\times C}$ and $y^{v}\in R^{W\times C}$. The output feature map is obtained by directly summing them:

(7) $$y_{c,i,j}=y_{c,j}^{h}+y_{c,j}^{v}$$

The output feature map is first subjected to a convolution operation and then activated by a sigmoid function. The result is fused with the original input feature map to obtain the final output. They are directly summed to obtain the output feature map:

(8) $$z=Scale(X,\sigma \left(f\left(y\right)\right))$$

In Equation (8), *Scale* denotes multiplication, σ represents the sigmoid function, and *f* indicates a 1 × 1 convolution operation.

#### 2.2.4. CGA-Based Mixup Fusion Scheme

**Figure 4:**
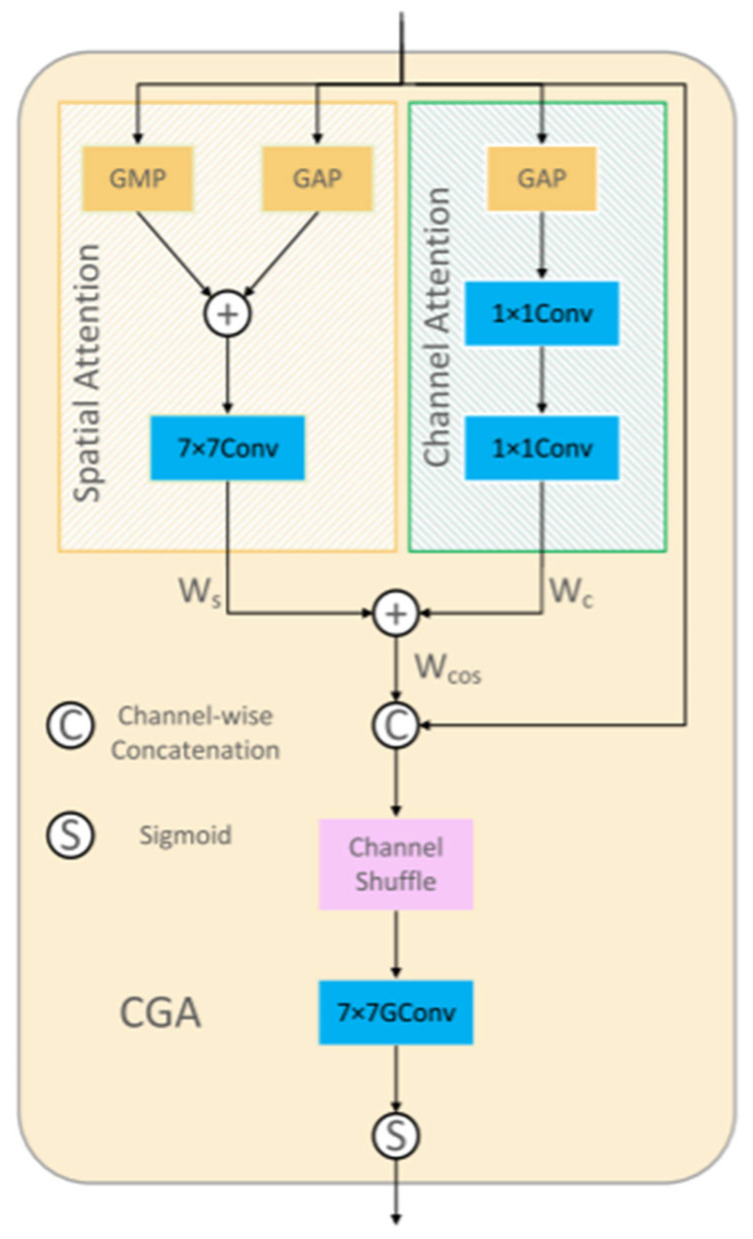
The structure of CGA.

The CGA-based mixup fusion scheme is an innovative feature fusion mechanism designed to address the inherent defects of traditional feature fusion methods in receptive field mismatch and information redundancy. The core of this scheme lies in dynamically generating channel-specific spatial importance maps (SIMs) to achieve fine-grained alignment and weight allocation for low-level and high-level features. Specifically, CGA achieves collaborative interaction through parallel channel attention and spatial attention, as shown in Figure 4, to first generate a preliminary global SIM. Subsequently, the content information of the input features is used to refine the SIMs at the channel level, enabling the SIM of each channel to adaptively reflect the key region distribution of that channel’s features. This two-stage design preserves global semantic information while enhancing the saliency of local details, providing more discriminative weight criteria for subsequent fusion.

For the input feature *X* ∈ *C* × *H* × *W*, the goal of CGA is to generate a SIM with the same dimension as *X*, that is, *W* ∈ *C* × *H* × *W*. First, $W_{c}$ and $W_{s}$ are calculated using the following equations:

(9) $$W_{c}=C_{1\times 1}(max(0,C_{1\times 1}(X_{GAP}^{c})))$$

(10) $$W_{s}=C_{7\times 7}(\left[X_{GAP}^{s},X_{GMP}^{s}\right])$$

where max⁡(0,x) is the ReLU activation function, $C_{k\times k}($.) represents a *k* × *k* convolutional, and [.] represents channel-wise concatenation. $X_{GAP}^{c}$, $X_{GAP}^{s}$, and $X_{GMP}^{s}$ represent the global average pooling operation across the channel dimension, the global average pooling operation across the spatial dimension, and the global max pooling operation across the spatial dimension, respectively. To reduce the number of parameters and limit the complexity of the model, the first 1 × 1 convolution reduces the channel dimension from *C* to *C*/*r* (where *r* is the reduction ratio), and the second 1 × 1 convolution expands it back to *C*. In the implementation of this paper, *r* is set to 8, reducing the channel dimension to a fixed value of *C*/8. Then, $W_{c}\;$ and ${\;W}_{s}\;$ are directly added according to the broadcasting rule to obtain

(11) $$W_{COS}\in \;C\times H\times W$$

To obtain the final *W*, each channel of ${\;W}_{COS}\;$ needs to be adjusted based on the corresponding input feature. Under the guidance of the input feature content, the final *W* is generated.

(12) $$W=\sigma (GC_{7\times 7}(CS([X,W_{COS}])))$$

where σ  represents the sigmoid operation, $CS\left(.\right)\;$ represents the channel-shuffling operation, and $GC_{k\times k}\left(.\right)\;$ represents a hierarchical convolutional group with a kernel size of *k* × *k*. CGA assigns a unique SIM to each channel, guiding the model to focus on the important regions within each channel. Therefore, more useful information encoded in the features is emphasized, effectively improving the performance.

**Figure 5:**
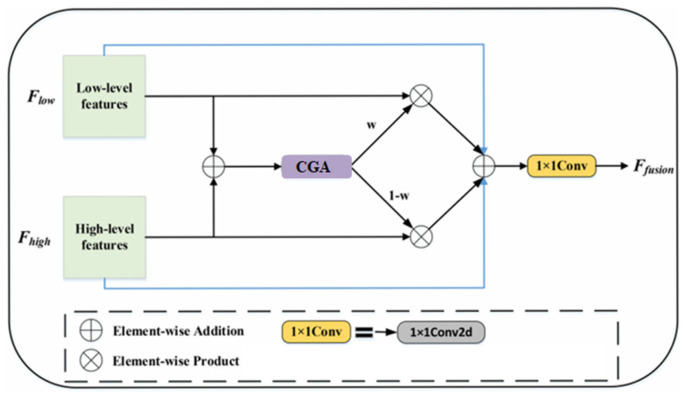
The structure diagram of the CGA-based mixup fusion scheme.

Compared with traditional direct concatenation or simple weighted fusion strategies, the CGA-based hybrid fusion scheme exhibits significant advantages. First, the concatenation operation assumes that features at different levels can be directly aligned spatially for concatenation. However, in practice, shallow features are limited by local receptive fields and often only capture texture details and edge information, while deep features form global semantic representations through multi-scale aggregation. These two types of features have fundamental differences in spatial coverage. Direct concatenation is prone to introducing high-frequency noise or diluting low-frequency information, making effective information interaction difficult. In contrast, the CGA-based scheme uses dynamically generated SIMs to spatially modulate features, explicitly modeling the receptive field differences between features at different levels. For example, in certain regions, CGA tends to assign higher weights to high-level semantic features to suppress noise, while in areas with clear edges, it enhances the contribution of low-level detail features, achieving adaptive feature selection and fusion. The structure diagram of the CGA-based mixup fusion scheme is shown in Figure 5.

The CGA-based mixup fusion scheme addresses the mismatch in receptive fields and information redundancy between high-level semantic and low-level spatial features. It employs a two-stage attention mechanism: first, parallel channel and spatial attention branches generate a global SIM by fusing global average/max pooling features. Second, input feature content guides channel-wise refinement of the SIM, enabling adaptive weight allocation for cross-scale feature interaction. By dynamically calibrating feature relevance through channel shuffling and hierarchical convolutions, this scheme enhances fine-grained alignment and suppresses redundant information.

### 2.3. Improved DeepLabv3+ Model

**Figure 6:**
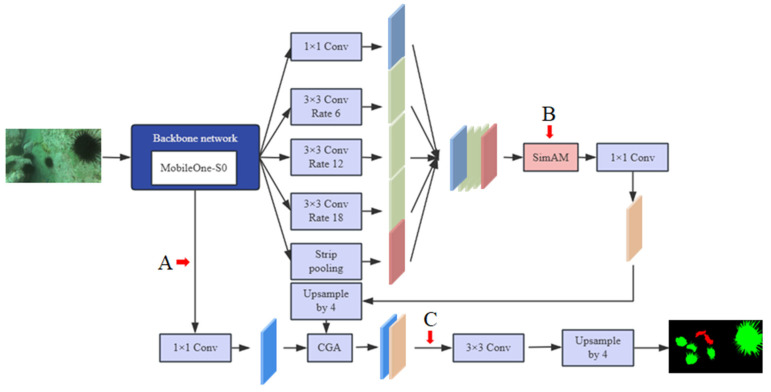
The structure of improved DeepLabv3+.

As shown in Figure 6, the input image first enters the feature extraction network with MobileOne-S0 as the backbone. Leveraging its unique structural reparameterization technology, MobileOne-S0 introduces linear branches during training and simplifies the structure during inference, effectively reducing memory access costs. This technology enhances model performance with low parameters, enabling the model to quickly and efficiently extract basic features of the image, providing a strong foundation for subsequent processing. After feature extraction by MobileOne-S0, the high-level features enter the ASPP module. ASPP captures multi-scale contextual information through dilated convolutions with different atrous rates. Now, SimAM is added after ASPP, followed by a 1 × 1 convolution. SimAM adaptively adjusts the weights of feature maps, highlights important features, suppresses irrelevant information, and enhances the model’s ability to capture target features in complex scenes, making the segmentation results more accurate. Global average pooling is replaced by strip pooling, which obtains richer global and local information through pooling operations in different directions. This addresses the problem of global average pooling possibly losing spatial information and further improves the model’s understanding of the overall image structure and details. In the feature fusion stage, the classic DeepLabv3+ uses the concatenation operation to fuse high-level and low-level features, while the improved model employs a CGA-based feature fusion scheme. By dynamically generating channel-specific SIMs, CGA achieves fine-grained alignment and weight allocation for shallow and deep features, enhancing interactions between features at different levels. This enables the model to better utilize the semantic and spatial information of images, thereby improving segmentation accuracy. The feature map processed as above is then upsampled to the original image resolution through bilinear interpolation and other operations to achieve pixel-level classification and obtain the final image segmentation result.

This improved DeepLabv3+ model combines the efficient feature extraction capability of MobileOne-S0, the attention mechanism of SimAM, the information acquisition advantages of strip pooling, and the CGA-based mixup feature fusion scheme, demonstrating superior performance in semantic segmentation tasks.

### 2.4. Loss Function

The Focal Loss function was initially proposed for object detection tasks to balance the contributions of easy and hard examples during training, enabling models to optimize parameters more effectively during backpropagation. The Dice Loss function, commonly employed in semantic segmentation, calculates loss by measuring the similarity between two samples, thereby mitigating the adverse effects of foreground–background class imbalance. In this study, the DUT-USEG dataset exhibits significant class imbalance between positive and negative samples, coupled with substantial variations in sample difficulty. To address these challenges, a hybrid loss function combining Focal Loss and Dice Loss was adopted to enhance the model’s segmentation performance. This approach ensures robust optimization under imbalanced data distributions while maintaining precise boundary delineation.

(13) $$Focal\;Loss=-{\left(1-p_{t}\right)}^{\gamma }\log\left(p_{t}\right)$$

where $p_{t}$ denotes the confidence score of the predicted class for a sample, while γ represents a tunable parameter with a default value of 2.

(14) $$Dice\;Loss=1-\frac{2\sum_{i=1}^{N}y_{i}\hat{y_{i}}}{\sum_{i=1}^{N}y_{i}+\sum_{i=1}^{N}\hat{y_{i}}}$$

where $y_{i}$ and $\hat{y_{i}}$ denote the ground truth value and predicted value of pixel i, respectively, and N represents the total number of pixels.

### 2.5. Model Training and Testing

#### 2.5.1. DUT-USEG Dataset

**Figure 7:**
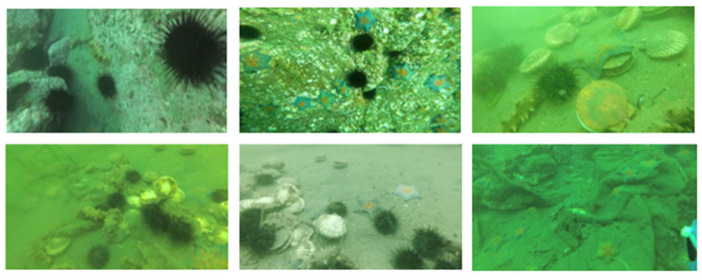
DUT-USEG dataset.

The DUT-USEG dataset, a real-world underwater semantic segmentation benchmark, comprises 6617 images of four marine species (echinus, holothurian, scallop, and starfish) with resolutions ranging from 586 × 480 to 3840 × 2160. It exhibits substantial class imbalance (e.g., 40,435 echinus instances vs. 1471 scallop instances), mirroring natural ecological distributions. For experiments, a subset of 1487 annotated images was utilized, partitioned into training, validation, and test sets at an 8:1:1 ratio, with input images resized to 512×512 pixels, as shown in Figure 7.

#### 2.5.2. Model Training Environment and Parameters

The experiments in this study were conducted on a Windows 10 64-bit operating system using PyTorch 1.12.1 and cuDNN 8.3.02 frameworks. The computational platform was equipped with an Intel Core i7-10700K processor, 16 GB of RAM, and an NVIDIA RTX A4000 GPU. The model was trained for 100 epochs with an initial learning rate of 1×10^−4^, where the learning rate was decayed by 10% every 10 epochs. A batch size of 8 and the Adam optimizer were employed during training, with a weight decay factor set to 0.0001 and other hyperparameters maintained at their default configurations.

#### 2.5.3. Model Evaluation Metrics

The evaluation metrics adopted in this study include the mean Intersection over Union (mIoU), mean Pixel Accuracy (mPA), number of trainable parameters, and floating-point operations (FLOPs). The specific equations of these metrics are defined as follows:

(15) $$mPA=\frac{1}{k+1}\sum_{i=0}^{k}\frac{p_{ii}}{\sum_{j=0}^{k}p_{ij}}$$

(16) $$mIoU=\frac{1}{k+1}\sum_{i=0}^{k}\frac{p_{ii}}{\sum_{j=0}^{k}p_{ij}+\sum_{j=0}^{k}p_{ji}-p_{ii}}$$

where $p_{ii}$ is the total number of pixel points that belong to class *i* and are predicted to be class *i*. $p_{ij}$ denotes the total number of pixel points that belong to class *i* but are predicted to be class *j*. $p_{ji}$ is the total number of pixel points that belong to category *j* but are predicted to be category *i*. *k* + 1 is the number of categories. mIoU evaluates global segmentation accuracy and boundary consistency. mPA quantifies pixel-level classification performance and inter-class recognition robustness. Parameters indicate model complexity and overfitting risks, whereas FLOPs assess computational efficiency.

## 3. Results and Discussion

### 3.1. Comparison of Different Models

To validate the efficacy of the proposed improved DeepLabv3+ algorithm, five semantic segmentation models, including UNet, PSPNet, HRNetv2, DeepLabv3+ (MobileNetV2), and SegFormer, were selected as comparative models for comprehensive performance analysis. To ensure the rigor and fairness of the experimental design, all comparative trials were conducted under strictly identical computational configurations and hyperparameter settings throughout the evaluation process. The results are presented in Table 2.

The comparative experiments demonstrate that the improved DeepLabv3+ model achieves an mIoU of 71.18% on the DUT-USEG dataset, outperforming UNet, PSPNet, HRNetv2, DeepLabv3+ (MobileNetV2), and SegFormer by 3.99%, 2.67%, 1.09%, 3.45%, and 2.81%, respectively. Furthermore, the proposed model attains an mPA of 80.42%, surpassing all comparative models and representing the sole model exceeding 80% in the comparative study. These results validate the effectiveness of the proposed improvements in enhancing semantic segmentation accuracy for underwater organisms.

In terms of lightweight performance, the improved DeepLabv3+ model exhibits a parameter value of 6.628 M, significantly lower than most comparative models, while slightly exceeding DeepLabv3+ (MobileNetV2) (5.814 M). Notably, the computational complexity of the proposed model, measured in FLOPs, is reduced to 39.612 G, achieving a 50.5% reduction compared to DeepLabv3+ (MobileNetV2) (79.949 G). Although the SegFormer model demonstrates marginally lower FLOPs (29.520 G), its parameters (44.605 M) remain substantially higher, underscoring the superior lightweight characteristics of the proposed architecture.

The improved model achieves an optimal balance between segmentation accuracy and computational efficiency. By maintaining minimal parameters, 6.628 M, while delivering excellent performance (71.18% mIoU, 80.42% mPA), the proposed method establishes a novel paradigm for underwater semantic segmentation tasks, addressing critical hardware constraints such as limited memory and computational resources in real-world marine exploration scenarios.

### 3.2. Ablation Study

The improved model is based on DeepLabv3+, with the backbone network replaced by MobileOne-S0, the SimAM applied to high-level features, strip pooling substituted for global average pooling, and the CGA-based mixup feature fusion used instead of concatenation. Ablation experiments were conducted using the same dataset and hyperparameters to validate the impact of these improvement strategies on the model’s segmentation accuracy and light weight, as shown in Table 3 and Table 4.

Replacing the backbone network of DeepLabv3+ from MobileNetV2 with MobileOne-S0 yields remarkable advantages. In terms of accuracy, both the mIoU and mPA are improved, enabling the model to more precisely identify and segment targets. Regarding lightweight performance, although the number of parameters slightly increases, the FLOPs are significantly reduced, leading to a decrease in inference computation and more efficient operation. This replacement achieves performance optimization, making the model more competitive in practical applications and providing strong support for semantic segmentation. Therefore, in the comparison of the ablation experiment results in Table 3 and Table 4, the backbone model of the proposed model is MobileOne-S0.

Table 4 presents the results of the ablation experiments conducted to evaluate the individual and combined effects of SimAM, strip pooling, and feature fusion on the model’s performance. When only SimAM was applied, mIoU increased by 0.58% and mPA increased by 0.35%. This indicates that SimAM can contribute to the model’s ability to better focus on relevant features, thus improving the segmentation accuracy to some extent. When CGA-based feature fusion was used alone, mIoU increased by 1.78% and mPA increased by 1.12%, which implies that the introduced feature fusion scheme can effectively enhance the model’s performance. Adding strip pooling while keeping SimAM results in an mIoU of 69.35% and an mPA of 79.89%. Although the improvement in mIoU is relatively small compared to the case with only SimAM, the mPA shows a slight increase, suggesting that strip pooling can have a positive impact on the pixel-level accuracy when combined with SimAM. Combining SimAM and feature fusion leads to an mIoU of 70.86% and an mPA of 80.06%, demonstrating a synergistic effect between the two methods. The experimental results demonstrate that the synergistic integration of these methods significantly enhances the model’s overall segmentation performance, providing critical insights into their individual contributions and interactive dynamics within the proposed architectural framework.

### 3.3. Comparison of Locations of SimAM Application

In this section, different control groups were also established. Considering that the attention module improves the adaptability of the convolutional network by suppressing irrelevant channel weights, experiments were conducted to explore the comparative effects of SimAM at the three common positions where the attention mechanism is added in the DeepLabv3+ network structure. The results are shown in Table 5, which is used to demonstrate the rationality of the position where SimAM is added in the DeepLabv3+ network structure.

The positions of A, B, and C are marked in Figure 6. A is the low-level features extracted by the backbone, SimAM, before the 1 × 1 convolution; B is SimAM after the ASPP and before the 1 × 1 convolution; C is SimAM after high-level and low-level feature fusion and before the 3 × 3 convolution. Applying SimAM to different positions does not necessarily enhance the semantic segmentation of underwater biological images. For example, in group 1, applying the attention module at position A reduced the model’s segmentation accuracy, possibly because the attention mechanism at this position disrupted the channel weights of the original network. Additionally, groups 4, 5, 6, and 7, which applied the SimAM module at multiple positions, exhibited inferior detection performance compared to group 2, which applied SimAM solely at position B. Therefore, SimAM was applied only after high-level feature fusion to improve the model’s detection capability for objects of various scales, thereby enhancing overall performance. This optimization increased the model’s mIoU to 69.12% and mPA to 79.49%.

### 3.4. Qualitative Analysis

**Figure 8:**
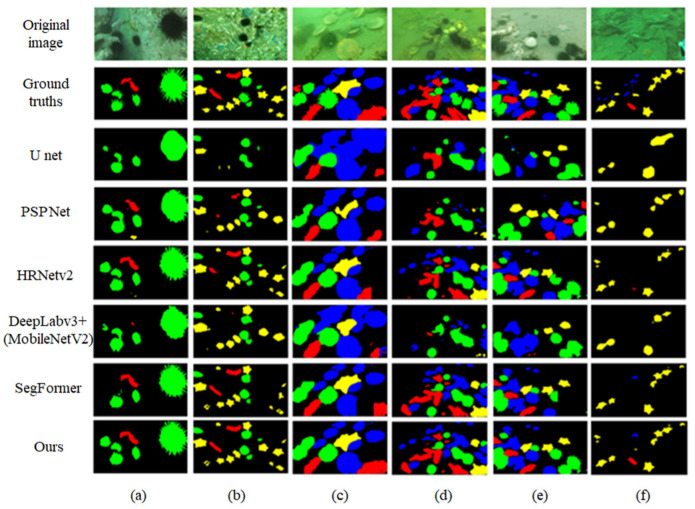
Segmentation results on the DUT-USEG dataset. (**a**–**f**) represent the segmentation results of six different images selected from the dataset.

In this section, to visually demonstrate the segmentation performance of the improved model, we selected several sample images from the DUT-USEG dataset for experimentation. Figure 8 compares the segmentation results of these sample images with other segmentation networks.

Qualitative analysis results demonstrate that the improved model exhibits outstanding segmentation performance. It can precisely delineate the edges of underwater organisms such as sea urchins, vividly presenting the fine details of sea urchin spines, and accurately segment small objects like sea cucumbers, thereby assisting underwater detection devices in identifying organisms and estimating their sizes. As shown in Figure 8a, the improved model not only completely segments all sea urchins but also clearly displays the minute spiny structures of the largest sea urchin, an achievement beyond the reach of other comparative models. In Figure 8f, due to the low contrast of the underwater environment and the tiny size of the sea cucumber, which is hard to see with the naked eye, only the improved model successfully accomplishes the segmentation task, while all other models fail to detect it. Compared with other comparative models, the improved model has a significant advantage in segmentation accuracy. It can accurately capture the fine-grained morphological features of underwater organisms, effectively mitigating the edge blurring caused by turbid water or low contrast. Additionally, it performs remarkably well in addressing the challenges of small underwater targets, such as vulnerability to noise interference and missed detection.

## 4. Conclusions

Aiming at the problems such as image blurring, indistinct target features, complex scenes, and limited performance of underwater hardware devices faced in underwater image semantic segmentation, a lightweight semantic segmentation model based on DeepLabv3+ is proposed. By integrating MobileOne-S0 as the backbone network, SimAM for high-level features, strip pooling for anisotropic context modeling, and a CGA-based mixup fusion scheme for efficient multi-scale feature integration, the proposed model achieves excellent performance on the DUT-USEG dataset, with an mIoU of 71.18% and an mPA of 80.42%. With only 6.628 M parameters and 39.612 G FLOPs, this work provides a practical solution for underwater exploration systems requiring a balance between segmentation accuracy and lightweight deployment.

The lightweight architecture of our model, achieving high accuracy with constrained computational demands, holds significant promise for real-world underwater exploration systems. Its efficiency enables seamless deployment on resource-constrained platforms such as AUVs, where low-latency, real-time semantic segmentation is critical for navigation and habitat mapping. Furthermore, the model’s portability facilitates integration into edge devices for collaborative marine research—for instance, empowering marine biologists to perform in situ species monitoring via compact underwater drones or fixed sensors, even in turbid or low-visibility environments. By bridging the gap between algorithmic innovation and practical deployability, our work advances scalable solutions for sustainable ocean exploration and ecological preservation.

Still, the research conducted in this paper does possess some deficiencies. Compared with some heavyweight semantic segmentation models, there is a certain gap in segmentation accuracy. However, this sacrifice in segmentation accuracy better meets the requirements of underwater hardware devices. In scenarios where the contrast is extremely low or the water is very turbid, the segmentation performance of this method is not ideal.

In the future, our research direction will focus on achieving higher accuracy while pursuing a more lightweight model. We will seek better improvement methods, such as incorporating multimodal data (e.g., sonar detection, thermal imaging information, etc.), performing more efficient feature extraction and fusion at multiple scales, and enhancing downsampling and upsampling methods. Additionally, we will attempt to apply the model to real-time semantic segmentation of underwater videos to adapt to dynamic and complex environments.

## Figures and Tables

**Table 1:** MobileOne network specifications.

Stage	Input	Blocks	Stride	Block Type	Channels	MobileOne Block Parameters (α,k, act = ReLU)				
						S0	S1	S2	S3	S4
1	224 × 224	1	2	MobileOne Block	64 × α	(0.75, 4)	(1.5, 1)	(1.5, 1)	(2.0, 1)	(3.0, 1)
2	112 × 112	2	2	MobileOne Block	64 × α	(0.75, 4)	(1.5, 1)	(1.5, 1)	(2.0, 1)	(3.0, 1)
3	56 × 56	8	2	MobileOne Block	128 × α	(1.0, 4)	(1.5, 1)	(2.0, 1)	(2.5, 1)	(3.5, 1)
4	28 × 28	5	2	MobileOne Block	256 × α	(1.0, 4)	(2.0, 1)	(2.5, 1)	(3.0, 1)	(3.5, 1)
5	14 × 14	5	1	MobileOne Block	256 × α	(1.0, 4)	(2.0, 1)	(2.5, 1)	(3.0, 1)	(3.5, 1, SE-RELU)
6	14 × 14	1	2	MobileOne Block	512 × α	(2.0, 4)	(2.5, 1)	(4.0, 1)	(4.0, 1)	(4.0, 1, SE-RELU)
7	7 × 7	1	1	AvgPool	-	-	-	-	-	-
8	1 × 1	1	1	Linear	512 × α	2.0	2.5	4.0	4.0	4.0

**Table 2:** Performance comparison of different models on the DUT-USEG dataset.

Model	mIoU (%)	mPA (%)	Parameters (M)	FLOPs (G)
Unet	67.19	78.34	43.933	184.200
PSPNet	68.51	79.68	46.708	369.481
HRNetv2	70.09	78.50	29.540	90.972
DeepLabv3+ (MobileNetV2)	67.63	77.62	5.814	79.949
SegFormer	68.37	78.26	44.605	29.520
Ours	71.18	80.42	6.628	39.612

**Table 3:** Comparative performance of backbone: MobileNetV2 and MobileOne-S0.

Backbone	mIoU (%)	mPA (%)	Parameters (M)	FLOPs (G)
MobileNetV2	67.63	77.62	5.814	79.949
MobileOne-S0	68.54	79.14	6.813	58.101

**Table 4:** Results of ablation experiments.

SimAM	Strip Pooling	Feature Fusion	mIoU (%)	mPA (%)
√			69.12	79.49
	√		68.97	80.22
		√	70.32	80.26
√	√		69.35	79.89
√		√	70.86	80.06
	√	√	70.35	80.13
√	√	√	71.18	80.42

**Table 5:** Effect of applying SimAM at different positions on the model.

Group	A	B	C	mIoU (%)	mPA (%)	Ascension of mIoU (%)
1	√			68.12	78.26	−0.42
2		√		69.12	79.49	0.58
3			√	68.89	79.03	0.35
4	√	√		68.61	78.93	0.07
5	√		√	68.22	78.37	−0.32
6		√	√	68.93	79.21	0.39
7	√	√	√	68.59	79.12	0.05

## Data Availability

The code for the data analysis is available upon request from the authors.
